# Endophytic *Aspergillus hiratsukae* mediated biosynthesis of silver nanoparticles and their antimicrobial and photocatalytic activities

**DOI:** 10.3389/fmicb.2024.1345423

**Published:** 2024-03-12

**Authors:** Ebrahim Saied, Mostafa A. Abdel-Maksoud, Akram A. Alfuraydi, Bushra Hafeez Kiani, Mohamed Bassyouni, Osama A. Al-Qabandi, Fathia H. E. Bougafa, Mona Shaban E. M. Badawy, Amr H. Hashem

**Affiliations:** ^1^Botany and Microbiology Department, Faculty of Science, Al-Azhar University, Nasr City, Egypt; ^2^Department of Botany and Microbiology, College of Science, King Saud University, Riyadh, Saudi Arabia; ^3^Department of Biology and Biotechnology, Worcester Polytechnic Institute, Worcester, MA, United States; ^4^Department of Chemical Engineering, Faculty of Engineering, Port Said University, Port Said, Egypt; ^5^Center of Excellence in Membrane-Based Water Desalination Technology for Testing and Characterization (CEMTC), Port Said University, Port Said, Egypt; ^6^College of Engineering and Technology, American University of the Middle East, Egaila, Kuwait; ^7^Department of Microbiology, Faculty of Science, Tobruk University, Tobruk, Libya; ^8^Department of Microbiology and Immunology, Faculty of Pharmacy (Girls), Al-Azhar University, Cairo, Egypt

**Keywords:** antimicrobial activity, biosynthesis, fungal endophytes, photocatalytic activity, silver nanoparticles

## Abstract

In the current study, endophytic *Aspergillus hiratsukae* was used for the biosynthesis of silver nanoparticles (Ag-NPs) for the first time. The characterizations were performed using X ray diffraction (XRD), Transmission electron microscopy (TEM), Scanning electron microscopy with energy dispersive X-ray spectroscopy (SEM–EDX), Dynamic light scattering (DLS), Fourier transform infrared spectroscopy (FT-IR), and UV–Vis spectroscopy. The obtained results demonstrated the successful formation of crystalline, spherical Ag-NPs with particle diameters ranging from 16 to 31 nm. The FT-IR studied and displayed the various functional groups involved, which played a role in capping and reducing agents for Ag-NPs production. The SEM–EDX revealed that the main constituent of the AS-formed sample was primarily Ag, with a weight percentage of 64.2%. The mycosynthesized Ag-NPs were assessed for antimicrobial as well as photocatalytic activities. The antimicrobial results indicated that the synthesized Ag-NPs possess notable antibacterial efficacy against *Staphylococcus aureus, Bacillus subtilis, and Escherichia coli*, with minimum inhibitory concentrations (MICs) of Ag-NPs ranging from 62.5 to 250 μg/mL. Moreover, the biosynthesized Ag-NPs demonstrated weak antifungal activity against *Aspergillus brasiliensis* and *Candida albicans*, with MICs of 500 and 1,000 μg/mL, respectively. In addition, the mycosynthesized Ag-NPs exhibited photocatalytic activity toward acid black 2 (nigrosine) dye under both light and dark stimulation. Notably, After 300 min exposure to light, the nigrosine dye was degraded by 93%. In contrast, 51% degradation was observed after 300 min in darkness. In conclusion, Ag-NPs were successfully biosynthesized using endophytic *A. hiratsukae* and also exhibited antimicrobial and photocatalytic activities that can be used in environmental applications.

## 1 Introduction

The exponential growth in population and global industrialization have created a huge demand for safe and drinkable water. Industrial effluents, dyes, fertilizers, pesticides, herbicides, and pharmaceutical wastes from a variety of industries, including textile, fertilizer, pharmaceutical, and petrochemical, as well as household operations, are the daily causes of huge water pollution. These pollutants also contaminate surfaces and groundwater (Ismail et al., 2019; Hanafi and Sapawe, 2020). Moreover, a significant amount of fertilizers, pesticides, and insecticides used to boost crop quality and yield but also polluted water are present in runoff water from agricultural land. If ingested, this chemically tainted water can cause severe, acute illnesses such as diarrhea, typhoid, hepatitis, skin rash, etc., and its prolonged consumption can be extremely harmful to human health (Zahoor and Mushtaq, 2023). Due to their chemical interaction with water molecules, textile dyes are among the organic contaminants that are the most difficult to remove from aqueous systems. Water pollution must be addressed seriously because it is the main cause of the spread of various diseases worldwide (Akhtar et al., 2021). Different classification systems for synthetic dyes are established based on the dye’s ionic charge in aqueous media, industrial application, chemical structure, and properties (Benkhaya et al., 2020). Most textile dyes are azo dyes, which comprise 65–75% of all textile dyes. They are characterized by the presence of an azo group (-N=N-) acting as a chromophore and the auxochrome group (-SO_3_H, sulphonate), which can be ionizable in aqueous media and gives the dyes their ability to bind to fibers. The three main categories of dyes are cationic dyes, nonionic dyes, and anionic dyes. Due to their affinity for forming covalent bonds with textiles and their greater brightness when compared to other dyes, anionic dyes are widely used in textile production (Benkhaya et al., 2020). A higher quantity of anionic dyes is therefore typically used. However, because of their positive surface charge and chemical characteristics used in smaller amounts because they have benign coloring and do not cause malignant tumors, while still being water-soluble they water-soluble, inhibit certain biological activities. Anthraquinone, xanthene, oxazine, cyanine, azine, acridine, azo, and certain anionic dyes including nitroso, nitro, triphenylmethane, anthraquinone, azo groups, etc. are examples of cationic dyes (Abdelaziz et al., 2023). Anionic and cationic dyes both cause cancer, but the risks connected to anionic dyes are more mutagenic and include bladder cancer, allergies, dermatitis, asthma, and other conditions. Dyes could also be categorized as non-ionic dyes in addition to the ionic division. These neutrally charged surface molecules are known as non-ionic dyes. It consists of two types of dyes: vat dyes, which react with leuco salts upon reduction in an alkaline medium and disperse dyes, which are water-insoluble and capable forming hydrophobic liquid dispersions (Shah H.U.R. et al., 2021). Disperse dyes are more carcinogenic in nature than vat dyes, which have very little toxicity. Indigoids, anthraquinone, nitro, azo, styryl, and other groups associated with chemical structures serve as examples of non-ionic dyes (Affat, 2021). Acid black 2 (nigrosine) is used as a dye for coloring fabrics such as silk, jute, cotton, and wool, as well as leather, plastic and wood; the manufacture of carbon paper; shoe polish cream; ink, toners, phenolic resins, styrenics, polyamides and urea resins; cosmetics; indicators, and color smoke (Sabnis, 2017). Typically, the presence of an azo group and aromatic rings serves to define nigrosine dye. These dyes’ complex aromatic chemical structures are thought to make them extremely harmful to living things and resistant to biodegradation (Homaeigohar, 2020). Due to their carcinogenic, mutagenic, allergy issues, vomiting, and cyanosis-causing qualities, they are also the ones causing the most worry (Karri et al., 2021). Due to these hazardous consequences, it is imperative to remove these colors from water bodies using a specific process in order to protect aquatic life (Khan et al., 2021). Due to size confinement, an increased surface area that enhances interfacial processes, and exhibit behaviors distinct from those of materials at the macroscale and have highly desired qualities (Baig et al., 2021). These specific properties of nanosized materials result in enhanced catalytic and adjustable photoactivity performances, higher strength, etc., making nanomaterials an important component for many different purposes. The use of nanotechnology in various approaches to environmental restoration is one of its most noteworthy applications (Ahmed et al., 2022; Ali et al., 2022; Hashem et al., 2022a; Hasanin et al., 2023). The synthesis of nanomaterials can be divided into two main categories: conventional methods and green methods. Conventional techniques for creating nanomaterials have a lot of enticing benefits. These methods produce a diverse array of nanoparticles with multiple applications. Novel applications for some of these approaches include battery conduction, electrical applications, targeted disease therapy, energy storage and conservation, extensive scalability, and precise control over nanoparticle morphology (Gour and Jain, 2019; Dikshit et al., 2021; Kumar et al., 2021). However, the obvious disadvantages of employing these outdated methods cannot be ignored. Because organic solvents are extensively used to synthesize these nanomaterials, there is a considerable risk to reproduction and neurobehavioral health (Huston et al., 2021). Hazardous working conditions may also result from applying high-pressure and hot conditions. The increased production of cardon dioxide, which significantly intensifies the greenhouse effect and raises concerns about volatile vapor, represents one of the most significant adverse effects of these syntheses. All things considered, these methods pose an irreversible risk to both the environment and the scientists performing the synthesis. The potential disadvantages of traditional methods for synthesizing nanomaterials outweigh their benefits. The decline in the popularity of traditional synthesis techniques has led to an increase in the popularity of green synthesis. Creating novel and forward-thinking methods that follow the Principles of green chemistry in light of the present climate catastrophe (Gour and Jain, 2019; Dikshit et al., 2021; Huston et al., 2021).

Green synthesis, also known as biosynthesis, is an inexpensive technique that is clean, safe, biocompatible, and environmentally friendly (Lashin et al., 2023; Shehabeldine et al., 2023). This process uses biological entities to reduce, cap, and stabilize metal or metal oxide precursors to form nanoparticles (NPs): bacteria, actinomycetes, yeast, fungi, algae, and plant extract (Dikshit et al., 2021; Albalawi et al., 2022; Hashem and El-Sayyad, 2023; Hashem et al., 2023c). Active metabolites, either secreted by microorganisms or found in plant extracts, facilitate the reduction and capping processes (Qamar and Ahmad, 2021). Proteins, amino acids, and enzymes are just a few of the many metabolites that fungi produce. These metabolites accelerate and improve the stability of nanoparticles produced in a green way. Due to their great stability, these metabolites are well known (Saied et al., 2023b). Moreover, fungi can produce NPs both intra- and extracellularly, are low in toxicity, easy to scale up, have good heavy metal accumulators, and are easy to handle (Rana et al., 2020; Abu-Elghait et al., 2021; Hashem et al., 2021). Endophytes are defined as organisms that live in tissues beneath the epidermal cell layers and have no obvious negative effects on the host (Yu et al., 2010; Hashem et al., 2023a). The most common microorganisms found as endophytes are fungi and bacteria. Fungi are the most commonly isolated endophytes. Fungal endophytes form a mutualistic or commensal relationship with their host plants. They colonize the intercellular spaces or live inside the plant cells, often within the roots, stems, leaves, or seeds (Alam et al., 2021). Fungal endophytes are used to synthesize silver nanoparticles (Netala et al., 2016; Guilger-Casagrande and Lima, 2019). Among the various types of metallic nanoparticles, silver nanoparticles are unique because they have a broad spectrum of antibacterial activity (Marathe et al., 2023). These nanoparticles may even penetrate bacterial cell walls and membranes to attach themselves. Cellular structures are damaged, signal transduction pathways are altered, and reactive oxygen species are generated (Agreles et al., 2022). Several studies have shown that silver nanoparticles may effectively suppress harmful bacteria in the medical and agricultural fields (Castillo-Henríquez et al., 2020; Tufail and Liaqat, 2021; Husain et al., 2023). Due to their unique biological, chemical, and physical properties, Ag-NPs are helpful in many fields, involving chemical catalysis, optoelectronics, biomedicine, and other. Owing to their toxicity and possible risks to human health and the environment, biogenic synthesis techniques are becoming more popular (Lekha et al., 2021; Raj et al., 2021). This study aimed to biosynthesize Ag-NPs using endophytic *Aspergillus hiratsukae* for the first time. Also, to evaluate their antimicrobial activity as well as the potential degradation of hazardous dyes in contaminated water.

## 2 Materials and methods

### 2.1 Chemical and reagents

Silver nitrate (AgNO_3_) and sodium hydroxide (NaOH) of 99% purity were utilized as analytical grade chemicals in the current work and were bought from Sigma Aldrich in Cairo, Egypt. Both the Mueller Hinton agar and Malt Extract agar (MEA) media, which were bought ready-made from Himedia in Cairo, Egypt, were employed for antibacterial activity. Distilled water (dis. H_2_O) was used to conduct all biological processes.

### 2.2 Green synthesis of Ag-NPs

Endophytic *Aspergillus hiratsukae* (Accession No. MT089951) was isolated from the leaves of *Avicennia marina* and identified morphologically and genetically, as detailed in our previous study (Khalil et al., 2021). In 100 mL of malt extract broth (MEB) medium, two disks of *A. hiratsukae* were inoculated and then incubated for 5 days at 28 ± 2°C with 150 rpm shaking. After the incubation period, the incubated MEB was centrifuged to extract the fungal biomass. The fungal biomass was resuspended for 48 h at 30 ± 2°C and 150 rpm shaking in 100 mL of distilled water. The previously mixed material was centrifuged at 10,000 rpm for 5 min to form AgNPs in a green manner. Fungal biomass filtrate was gathered and used (Al-Soub et al., 2022). The final concentration of the solution was 4.0 mM after mixing 100 mL of cell-free filtrate with silver nitrate. The mixture was left in the dark for 24 h at 28°C ± 2°C, with the pH adjusted to 10. Once the filtrate was dark brown, it was collected and rinsed with deionized water to remove any remaining impurities before being oven-dried for 8 h at 200°C. The experiment was conducted under the same conditions as the controls, which included AgNO_3_ solutions and fungal biomass filtrate (Hassan et al., 2021).

### 2.3 Characterization of Ag-NPs

UV–Vis analysis was carried out to measure absorbance between 300 and 800 nm using a UV–Vis spectrophotometer (JENWAY 6305 spectrophotometer). The size and shape of Ag-NPs were characterized using transmission electron microscopy (TEM) (JEM1230, Japan, Akishima, Tokyo, 196–8558) (Hashem et al., 2023b). SEM–EDX was used to analyze the elemental compositions of Ag-NPs produced during biosynthesis. The crystalline structure of Ag-NPs was determined using X-ray diffraction (XRD) analysis performed with an X’Pert Pro diffractometer (Philips, Eindhoven, Netherlands). The values of 2θ were measured between 4° and 80°. The Debye–Scherrer equation was used to calculate the average size of Ag-NP (Thakar et al., 2022). Furthermore, the size distribution of Ag-NPs in the colloidal solution was analyzed using dynamic light scattering (DLS) analysis. The material was tested using a tiny scattering spectrometer from Malvern Instruments Ltd. in Worcestershire called the Malvern Zetasizer Nanoseries. The polydispersity index (PDI) considers the homogeneity of the NPs solutions (Soliman et al., 2021). Alternatively, the functional groups associated with the stability, capping, and reduction of Ag-NPs in the fungal biomass filtrate were investigated using Fourier transform infrared (FT-IR) spectroscopy (Agilent System Cary 660 FT-IR Model). The 400–4,000 cm^−1^ range was used for the scanning.

### 2.4 Antimicrobial activity

The antimicrobial activity of Ag-NPs, silver nitrate, and fungal extract (FE) was evaluated against *Escherichia coli* ATCC 25922, *Bacillus subtilis* ATCC 6051, *Staphylococcus aureus* ATCC 25923, *Candida albicans* ATCC 90028, and *A. brasiliensis* ATCC 16404 using the agar well diffusion method. Bacterial suspensions with a concentration of 1.5 × 10^7^ CFU/mL were individually prepared. These suspensions were inoculated into Muller Hinton agar media and aseptically poured into sterilized petri plates. The fungal suspensions were evenly dispersed onto agar Potato Dextrose Agar (PDA) plates. In each plate, four agar wells with a diameter of 8 mm were created using a cork borer, then 100 μL of Ag-NPs, silver nitrate, FE (fungal extract), SAM (Ampicillin/sulbactam)/ Fluc (Fluconazole) at concentrations of 1,000 μg/mL were transferred to wells separately, and incubated for 24/72 h at 37/30°C for bacterial and fungal strains, respectively. After that, the inhibition zone diameters were measured. Different concentrations of Ag-NPs, silver nitrate, and FE (1,000, 500, 125, 62.5, 31.25, 15.62, 7.8, and 3.9 μg/mL) were used to detect MIC (Valgas et al., 2007).

### 2.5 Decolorization of acid black 2 (nigrosine) dye by Ag-NPs

The decolorization of nigrosine dye by biosynthesized Ag-NPs was studied at different concentrations (100, 150, and 200 mg/mL) over various contact times (30, 60, 120, 180, 240, and 300 min). The test was carried out in a 250 mL conical flask with 100 mL of 100 ppm nigrosine dye mixed with various Ag-NPs concentrations. The identical responses in a different portion of the experiment were carried out in the dark for a comparison examination. Prior to the experiment, the liquid was agitated for 30 min to achieve equilibrium between absorption and desorption. After each incubation period, 1.0 mL of the combination (nigrosine dye and NPs) was removed, centrifuged for 7 min at 5,000 rpm, and its optical density was assessed using a spectrophotometer (721 spectrophotometers, M-ETCAL) to determine its optical density at the nigrosine dye’s maximum absorption band (λmax) (570 nm) (Kumawat et al., 2019). The decolorization percentages (%) of nigrosine dye were calculated according to the following equation:


$$\text{Decolorization percentages}(\%)=\frac{C0-Cf}{C0}\times 100.$$

where C0 is the absorbance at zero time and *Cf* is the absorbance after a specific time t (min). To boost the dye adsorption even further, the catalyst’s reusability was examined through five cycles. The nano-catalyst was used in the second cycle after being retrieved from the first cycle and having undergone three washes with distilled water to eliminate any leftover water.

### 2.6 Statistical analysis

The means of three separate replications were used to calculate all the findings. A statistical tool called SPSS v.17 was used to statistically analyze the data. With a *p*-value of 0.05 or less, the mean difference between the treatments was compared using the Tukey HSD test.

## 3 Results and discussion

### 3.1 Biosynthesis and characterization of Ag-NPs

The green synthesis of silver nanoparticles has various benefits compared to alternative synthesis methods, such as reduced manufacturing costs, decreased environmental pollution, lower toxicity, and enhanced biological compatibility (Nangare and Onkar, 2020). Ag-NPs generated biologically have been shown to exhibit intriguing antibacterial and photocatalytic capabilities in recent years (El-Ansary et al., 2023; Omran, 2023). The results of this study revealed that metabolites of *A. hiratsukae* can biosynthesize Ag-NPs, leading to enhance production, reduce aggregation, and yield smaller-sized AgNPs (Alfryyan et al., 2022). Aspergilli have the ability to synthesize various NPs such as Ag-NPs. The presence of metal precursors affected the color of the biomass filtrate, which was the first sign that NPs were being biosynthesized. Saied E. et al. (2023) utilized the extracellular extract of *Rhizopus oryzae* to synthesize Ag-NPs. Additionally, Ahmed and Dutta (2019) synthesized the AgNPs utilizing the supernatants of *Trichoderma asperellum*. Saied et al. (2022a) successfully synthesized AgNPs using *Cytobacillus firmus*. According to Eid et al. (2020), *Streptomyces laurentii* biomass was used to biofabricate Ag-NPs. Furthermore, several studies focused on the biosynthesis of AgNPs using different biological extractions, like plant extract or microbial media (Shah M.Z. et al., 2021; Hamed and Kelany, 2023). Additionally, Moges and Goud (2022) created Ag-NPs by using methanol and successive aqueous extracts of the underutilized berries and leaves of *Hippophae salicifolia*, which is grown in northeast India.

**Figure 1:**
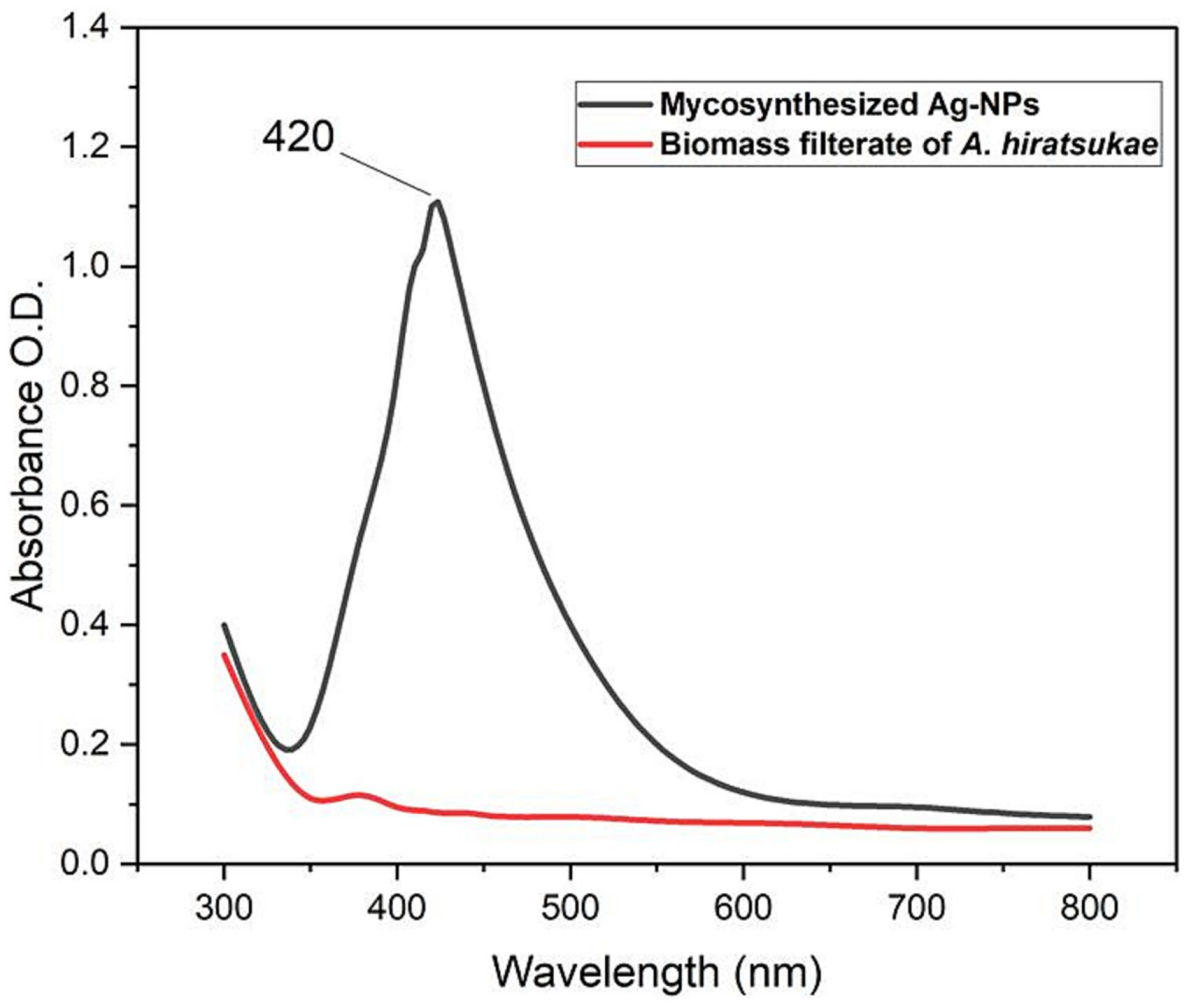
UV–Vis absorption spectra of silver nanoparticles synthesized by *A. hiratsukae*.

Ag-NPs have been characterized using physiochemical analysis and topographical examinations. Figure 1 shows the UV–visible spectra of Ag-NPs. The first indication of Ag-NPs production is a change in color to deep brown. The UV–Vis spectrophotometer investigation provided preliminary confirmation for Ag-NPs production. Variations in color intensity might be attributed to the surface plasmon resonance excitation of the synthesized nanoparticles (Abo-Elmagd et al., 2022). The production of Ag-NPs through mycosynthesis was confirmed when a peak at 420 nm was detected in the Ag-NPs spectra. A similar observation was made by Balaraman et al. (2020), who identified a distinct peak of Ag-NPs at 420 nm by using the marine algae *Sargassum myriocystum*. Dara et al. (2020) synthesized a Chi-Ag-NPs composite and exhibited a peak at 419 nm. Alwhibi et al. (2021) reported that Ag-NPs’ plasmon absorbance peaked at 450 nm. Conversely, higher maximum wavelength values are linked to lower Ag-NP concentrations, while lower average Ag-NP sizes are linked to higher concentrations (Awad et al., 2022). Alkhathlan et al. (2020) reported that GE-Ag-1 showed an absorption peak at 430 nm, while NSE-Ag-1 showed an absorption peak at 410 nm. The quality of the resulting NPs can be ascribed to the minor variation (~20 nm) in the positions of the UV peaks of AgNPs prepared using low concentrations of GE and NSE extracts.

**Figure 2:**
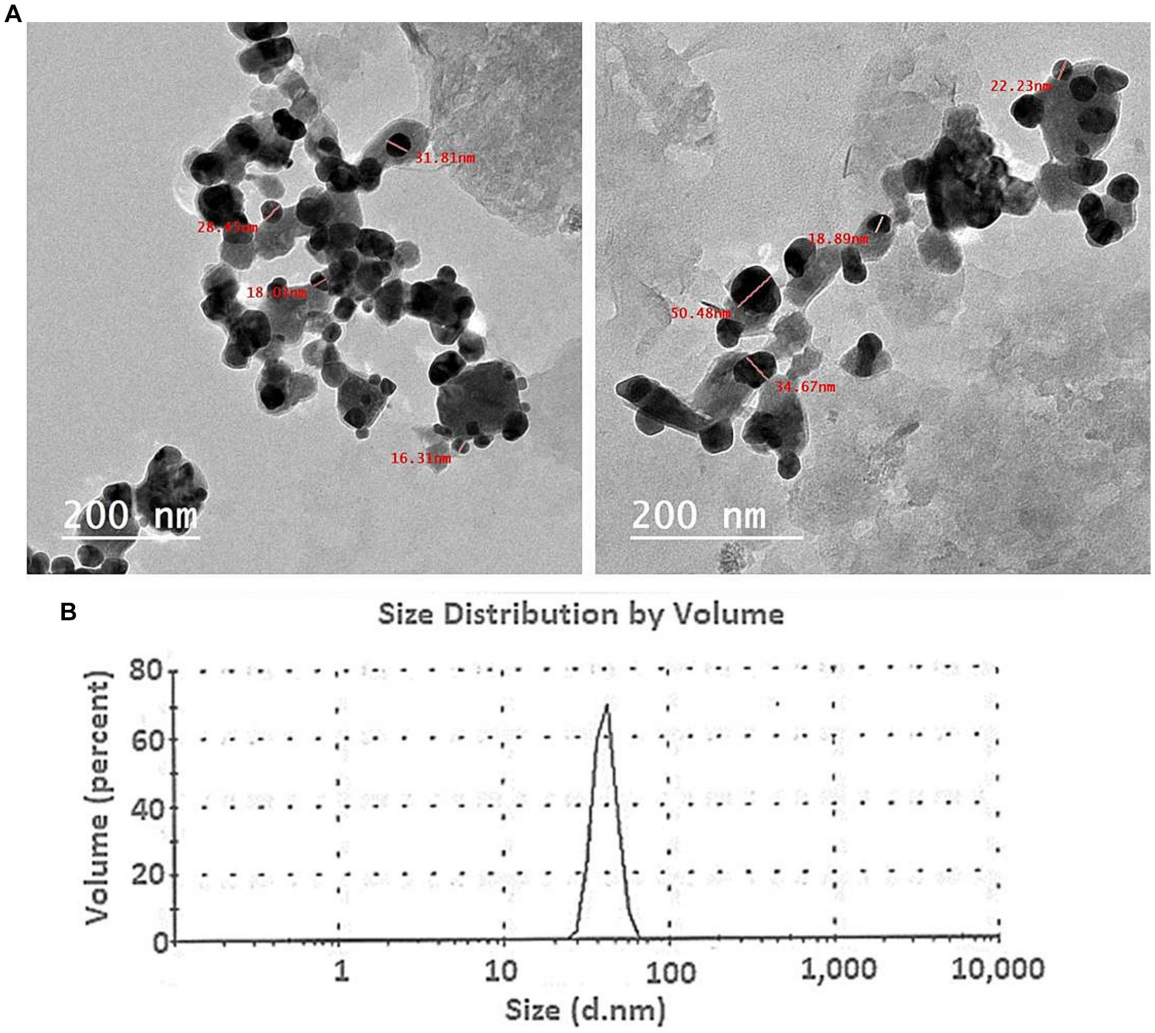
**(A)** The TEM image of the Ag-NPs produced by *A. hiratsukae* revealed a spherical shape, and **(B)**, the DLS analysis revealed the size distribution.

The TEM data provided insightful information about the morphology, dimensions, and distribution of the Ag nanoparticles produced through biological methods. The TEM analysis showed that the Ag-NPs produced with the mycelial-free filtrate of *A. hiratsukae* were spherical in shape and varied in size, ranging from 16 to 31 nm. The nanoparticles were evenly distributed, and there was no indication of particle aggregation or clumping, as shown in Figure 2A. Saied E. et al. (2023), who created Ag-NPs, reported the same outcome and discovered they were spherical with a 17–35 nm diameter. Alharbi and Alsubhi (2022), synthesized Ag-NPs with TEM imaging that were spherical with diameters of 27 nm. Based on a study conducted by El-fallal et al. (2022), the TEM analysis revealed that the AgNPs synthesized by Agrocybe cylindracea exhibited a spherical shape and a range of sizes from 3.47 to 13.99 nm. In a recent study by Nguyen et al. (2020), it was found that the P.uri. AgNPs exhibited a range of sizes, from 4 to 52 nm, and predominantly had a spherical and oval shape. Additionally, Fouda et al. (2022) synthesized Ag-NPs with spherical shapes, varying in size from 3 to 28 nm, with an average diameter of 12.5 ± 5.1 nm. Ag-NPs generated by the stem barks of the medicinal *Pyrus pashia* plant were analyzed for size and shape using TEM by Khanal et al. (2023). The average size of Ag-NPs was found to be 23.92 ± 7.04 nm, and they were found to be spherical and polydispersed. The compounds found in *A. hiratsukae* extract may be used to biosynthesize Ag-NPs with unique structures. Furthermore, the modest size of Ag-NPs generated in this study presents an opportunity for a range of size-dependent biotechnological applications.

The DLS analysis was employed to ascertain the size and particle distribution of NPs in colloidal solutions, much like a microbiologist would do. Scattering intensities from time-dependent data can be used to determine the hydrodynamic diameter, just as a microbiologist would do. Understanding the role of capping and stabilizing agents and the electrical layers on nanoparticle surfaces, is essential for controlling the hydrodynamic diameter of these particles (Divyalakshmi and Thoppil, 2023). DLS is more appropriate for early-stage aggregation monitoring because of its sensitivity to the presence of aggregates. The DLS analysis conducted in this study yielded an average size of 72 nm (70% volume) for the biosynthesized Ag-NPs (Figure 2B). According to Hashem et al. (2022b), the biosynthesized Ag-NPs had an average size of 32.7 nm and a size distribution histogram from 30 to 47 nm. The average size of the biosynthesized Ag-NPs, according to Saied E. et al. (2023), was 78 nm. The average diameter sizes of Ag-NPs and AgNPs-cis, using dynamic light scattering analysis, according to Alharbi and Alsubhi (2022), were found to be 249 and 260 nm, respectively. The polydispersity index (PDI) value was used to determine whether the colloidal NPs were homogeneous or heterogeneous (Soliman et al., 2021). A homogeneous solution is characterized by a PDI number less than 0.4; while a value greater than 0.4 signifies a heterogeneous solution, reflecting the degree of homogeneity. During our investigation, a PDI value of 0.163 was recorded, denoting a high level of homogeneity.

**Figure 3:**
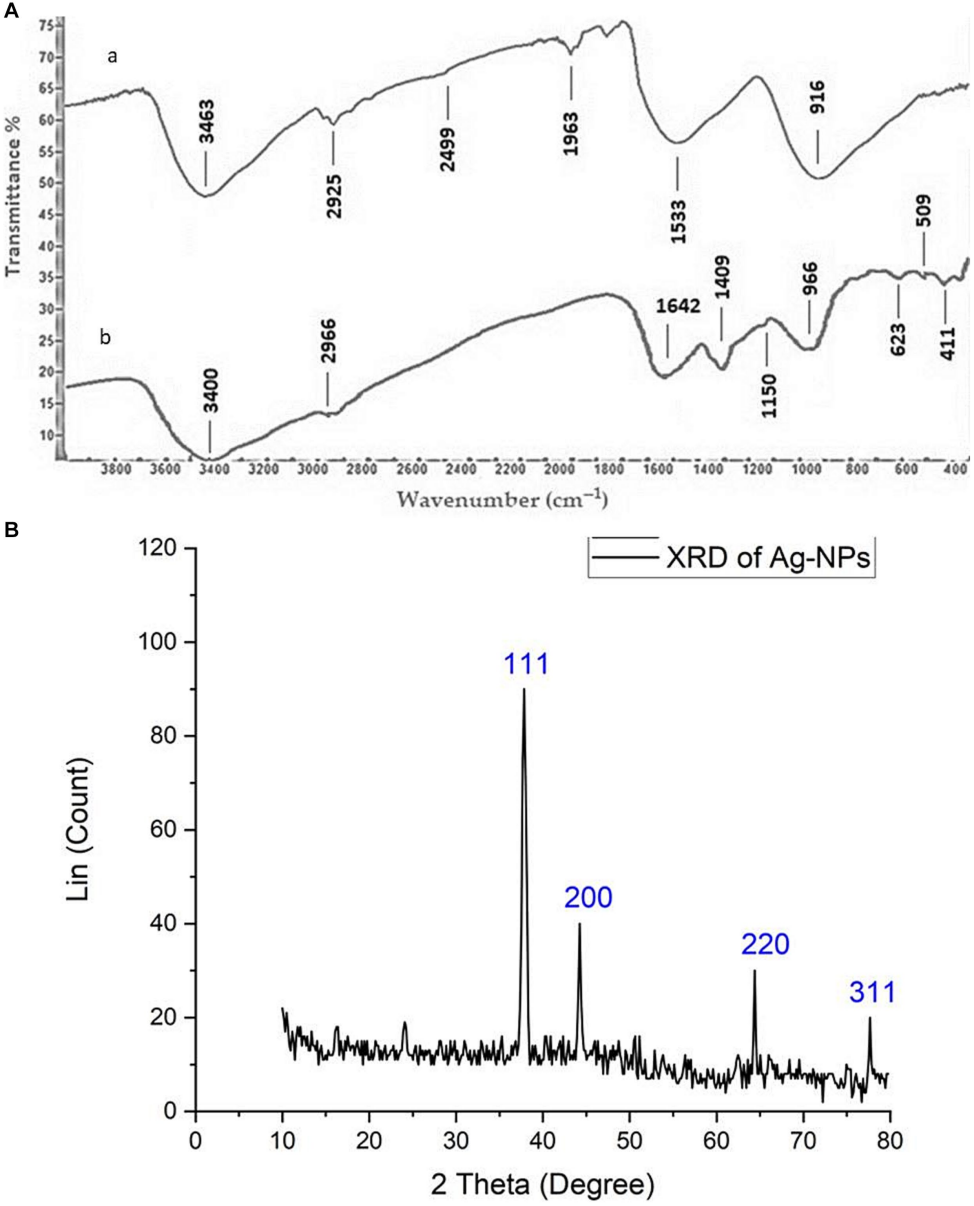
**(Aa,b)** FT-IR chart showing Ag-NPs made from *Aspragillus hiratsukae* and extra tact of the mycelial-free filtrate of *A. hiratsukae*. **(B)** Analysis of AgNPs using X-ray diffraction (XRD).

FT-IR spectroscopy is commonly employed in the analysis of biomolecules that encompass nanoparticles and serve as capping and stabilizing agents. The filtrate derived from fungal biomass comprises proteins and enzymes that play a crucial role in the stability and production of nanoparticles (Li et al., 2021). FT-IR analysis was utilized to explore the interaction between silver nanoparticles (AgNPs) and the supernatant of *A. hiratsukae*, as illustrated in Figure 3Ab. FT-IR spectra of the Ag-NPs synthesized through biosynthesis exhibited notable absorption peaks at 3,400, 2966, 1,642, 1,409, 1,150, 966, 623, 509, and 411 cm^−1^ wavenumbers. Figure 3Aa displays the FT-IR spectra, which exhibit prominent absorption peaks at 3,463, 2,925, 2,499, 1,963, 1,533, and 916 cm^−1^. The peaks observed at 3,400 and 3,463 cm^−1^ wavenumbers can be attributed to the O-H stretching groups present in phenols and alcohols, and the N-H groups found in amino acids inside proteins, respectively (Fouda et al., 2022). The peaks detected at 2,965 and 2,925 cm^−1^ indicate the vibrational stretching of C–H bonds in alkanes (Nefri and Djamaan, 2020). Nevertheless, the N-H bending of amines coincided with the peak observed at 1,640 cm^−1^, which was attributed to the stretching vibrations of C=O and C=N bonds. The presence of C-N stretching in aromatic and aliphatic amines is evidenced by the peak observed at 1,409 cm^−1^ (Iqbal et al., 2022). The peak seen at 1,150 cm^−1^ can be attributed to the asymmetric stretching of the C–O–C bond, as well as the stretching of the C–O bond and the rocking motion of the NH_2_ group in the polysaccharide groups (Al-Soub et al., 2022). The 1,533 cm^−1^ peak is associated with the stretching of the C=O bond in carboxylate salt, as well as the adsorption of CO_3_^2−^ and CO_2_ (Hashem et al., 2022b). Amide IV (OCN) stretch bending in proteins was identified through spectral peaks observed at wavenumbers of 623, 916, and 966 cm^−1^ (de Souza and Rodrigues, 2015). The protein stretch band was observed at a wavenumber of 509 cm^−1^ (Salem et al., 2022). The calcinated Ag-NPs exhibited a peak at a wavenumber of 411 cm^−1^ (Saied E. et al., 2023). The data identified and confirmed several functional groups, including alkanes, alkenes, aliphatic and aromatic amines, and alkyls. The compounds mentioned above have a crucial role in the stabilization, capping, and reduction of Ag-NPs, and they may be identified within the cell-free filtrate of *A. hiratsukae*. These results are consistent with the findings of El-fallal et al. (2022), on *Agrocybe Cylindracea* and *Agrocybe aegerita* by Nagalakshmi (2015).

Figure 3B illustrates the X-ray diffraction (XRD) pattern of the Ag-NPs synthesized by a biological process. A total of four discrete peaks were seen at certain 2θ angles: 37.84°, 44.3°, 64.1°, and 77.56°. The peaks seen in the data correspond to the crystal planes (111), (200), (220), and (311), respectively. This suggests a favorable alignment between the formation of Ag-NPs and the crystalline phase of silver (Balaji et al., 2023; Saied E. et al., 2023). The detected peaks for various 2θ values exhibited a face-centered cubic morphology. Significantly, the distinct peak at 2θ = 37.8° (111) showed a considerable degree of alignment with the investigated facet (111), indicating a high purity level in the synthesized Ag-NPs. The average size of nanoparticles was determined using the Debye–Scherrer equation, based on the X-ray diffraction (XRD) data. The average size of the AgNPs was 37.96 nm, while the full width at half maximum (FWHM) value of their 2θ peak was measured to be 0.23111. In line with our observations, Nirmala and Sridevi (2021) reported that the average diameter of the nanoparticles synthesized from endophytic bacteria was measured to be 16.8 nm. Additionally, Kareem et al. (2019) show five intense peaks in the X-ray diffractogram of the AgNPs synthesized using *A. alternata*. Sudarsan et al. (2021). determined that the mean size of the synthesized nanoparticles derived from the endophytic bacteria *Cytobacillus firmus* was 14.23 nm. AgNPs displayed a crystalline face-centered cubic (fcc) structure with an average size of approximately 49.3 nm (Sharma et al., 2022).

**Figure 4:**
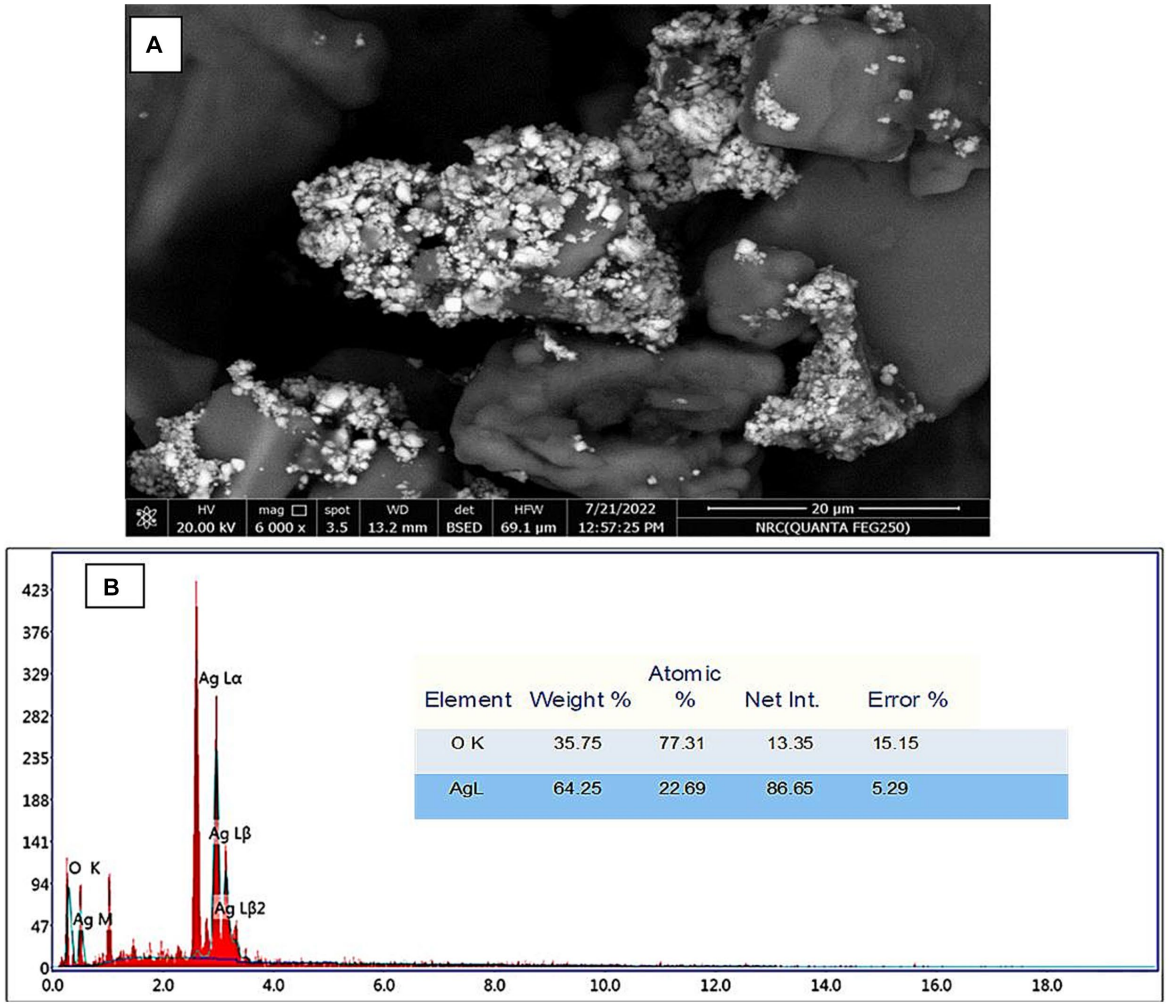
**(A)** SEM image of AgNPs. **(B)** EDX spectrum of the mycosynthesized Ag-NPs.

The morphologies of Ag-NPs formed during biosynthesis were analyzed using SEM. Figure 4A displays the results of the Scanning Electron Microscope (SEM) analysis conducted on the silver nanoparticles that were synthesized using a biological process. The findings of this study offer evidence of the nanoparticles’ small dimensions and spherical morphology, as identified through X-ray diffraction (XRD) analysis. Scanning electron microscopy reveals the presence of aggregate structure inside the powder particles. The observation was made that Ag-NPs tended to aggregate into small clusters, ultimately resulting in the formation of bigger particles. The outcome of this experiment yielded Ag-NPs that were consistent with prior research findings (Nirmala and Sridevi, 2021; M et al., 2022; Saied E. et al., 2023). Due to their excellent electrical conductivity, metal nanoparticles such as gold and silver can be easily examined using a SEM. SEM cannot analyze the internal structure of materials. However, it can provide valuable information on the integrity and aggregation of particles (Osterberg et al., 2023). The EDX profile of the Ag-NPs, Figure 4B, indicates the presence of the Ag element. The Ag element is present at a 22.6% atomic percentage. Ag accounts for 64.2% of the total weight, while O makes up 35.7%. O has the greatest atom percentage (77.3%). According to Hikmet and Hussein (2021), the EDX analysis revealed that the sample contained a high weight percentage of silver (82.4%). Additionally, the graph of EDX displayed signals corresponding to atoms of gold, carbon, oxygen, and chlorine.

### 3.2 Antimicrobial activity

**Figure 5:**
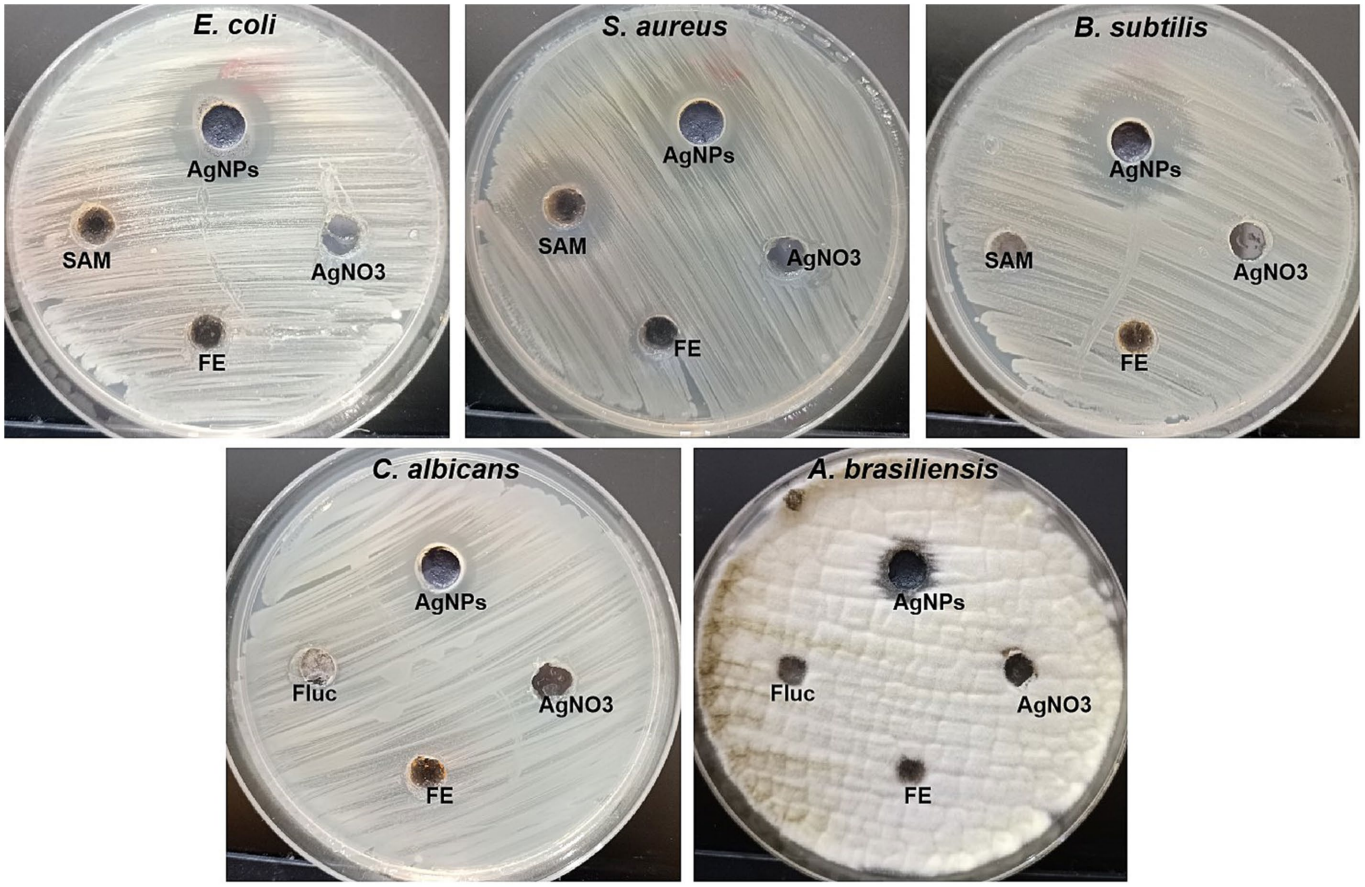
Antibacterial and antifungal activity of Ag-NPs, AgNO_3_, Fungal extract (FE) and SAM toward *E. coli, S. aureus, B. subtilis, C. albicans*, and *A. brasiliensis* using agar well diffusion method.

The antimicrobial efficacy of silver nanoparticles has been evaluated for the control of both multidrug-resistant (MDR) and non-MDR variants of bacteria, fungi, and viruses In the current study, the antimicrobial activity of mycosynthesized Ag-NPs was assessed, as shown in Figure 5 and Table 1. Results revealed that the mycosynthesized Ag-NPs exhibited promising antibacterial activity against all bacterial tested strains compared to the standard antibiotic Ampicillin/sulbactam (SAM). The inhibition zones of Ag-NPs toward *E.coli, S. aureus* and *B. subtilis* were 19.3 ± 1.5, 14.9 ± 1.1, and 22.0 ± 1.0 mm, respectively. Furthermore, antibacterial results showed that both *E.coli* and *B. subtilis* were the most sensitive to Ag-NPs where the MIC was 62.5 μg/mL for each one, while as *S. aureus* was the least sensitive, where the MIC was 250 μg/mL. On the other hand, fungal extract (FE) and AgNO_3_ did not give any inhibition against all tested strains except *S. aureus* which was weakly sensitive to FE where the inhibition zone was 10.2 ± 1.4 mm. As well, Ag-NPs showed weak antifungal activity toward unicellular and multicellular fungi, where inhibition zones and MIC were (12.2 ± 1.4 and 10.3 ± 0.6 mm) and (500 and 1,000 μg/mL) against *C. albicans* and *A. brasiliensis, respectively.* Conversely, both FE and AgNO_3_ exhibited no activity toward *C. albicans* and *A. brasiliensis*. Previous studies reported that endophytic fungi could biosynthesize Ag-NPs, which are used for different biological applications (Hu et al., 2019; Sharma et al., 2022). Netala et al. (2016) used *Aspergillus versicolor* ENT7 for the mycosynthesis of Ag-NPs, which exhibited promising antibacterial as well as antifungal activity. Ahluwalia et al. (2014) synthesized Ag-NPs using *Trichoderma harzianum*, which showed antibacterial activity against *S. aureus* and *Klebsiella pneumoniae*. Balakumaran et al. (2015) demonstrated the efficacy of the potential of silver nanoparticles synthesized using the fungus Guignardia mangiferae in controlling gram-negative bacteria The effects observed of Ag-NPs included increased permeability, modification of membrane transport, and the release of nucleic acids.

**Table 1:** Inhibition zones and MIC of mycosynthesized AgNPs and other start materials.

Fungal strain	AgNPs		FE		AgNO_3_		SAM/Fluc	
	IZ	MIC	IZ	MIC	IZ	MIC	IZ	MIC
*E. coli*	19.3 ± 1.5^a^	62.5	ND	ND	ND	ND	10.5 ± 0.9^b^	1,000
*S. aureus*	14.9 ± 1.1^b^	250	10.2 ± 1.4^a^	1,000	ND	ND	19.5 ± 1.2^a^	125
*B. subtilis*	22.0 ± 1.0^a^	62.5	ND	ND	ND	ND	ND	ND
*C. albicans*	12.2 ± 1.4^bc^	500	ND	ND	ND	ND	ND	ND
*A. brasiliensis*	10.3 ± 0.6^c^	1,000	ND	ND	ND	ND	ND	ND

FE, SAM and Fluc mean Fungal extract, Ampicillin/sulbactam and Fluconazole. Letters a, b, c means significance power.

The antimicrobial properties of Ag-NPs are associated with four distinct mechanisms. To begin with, it should be noted that silver nanoparticles tended to cling to both the cell wall and membrane. Furthermore, silver nanoparticles can infiltrate cellular structures and induce harm to several intracellular components, including mitochondria, vacuoles, and ribosomes, as well as biomolecules such as proteins, lipids, and DNA. Furthermore, the cellular toxicity and oxidative stress induced by Ag-NPs can be attributed to their ability to generate reactive oxygen species (ROS) and free radicals. Additionally, Ag-NPs possess the ability to modulate signal transduction pathways. In addition to those above four established methods, silver nanoparticles also exert influence on the immune system of human cells by regulating the inflammatory response, thus contributing to the suppression of microbes (Tian et al., 2007).

### 3.3 Ag-NPs-mediated photocatalytic nigrosine dye degradation

**Figure 6:**
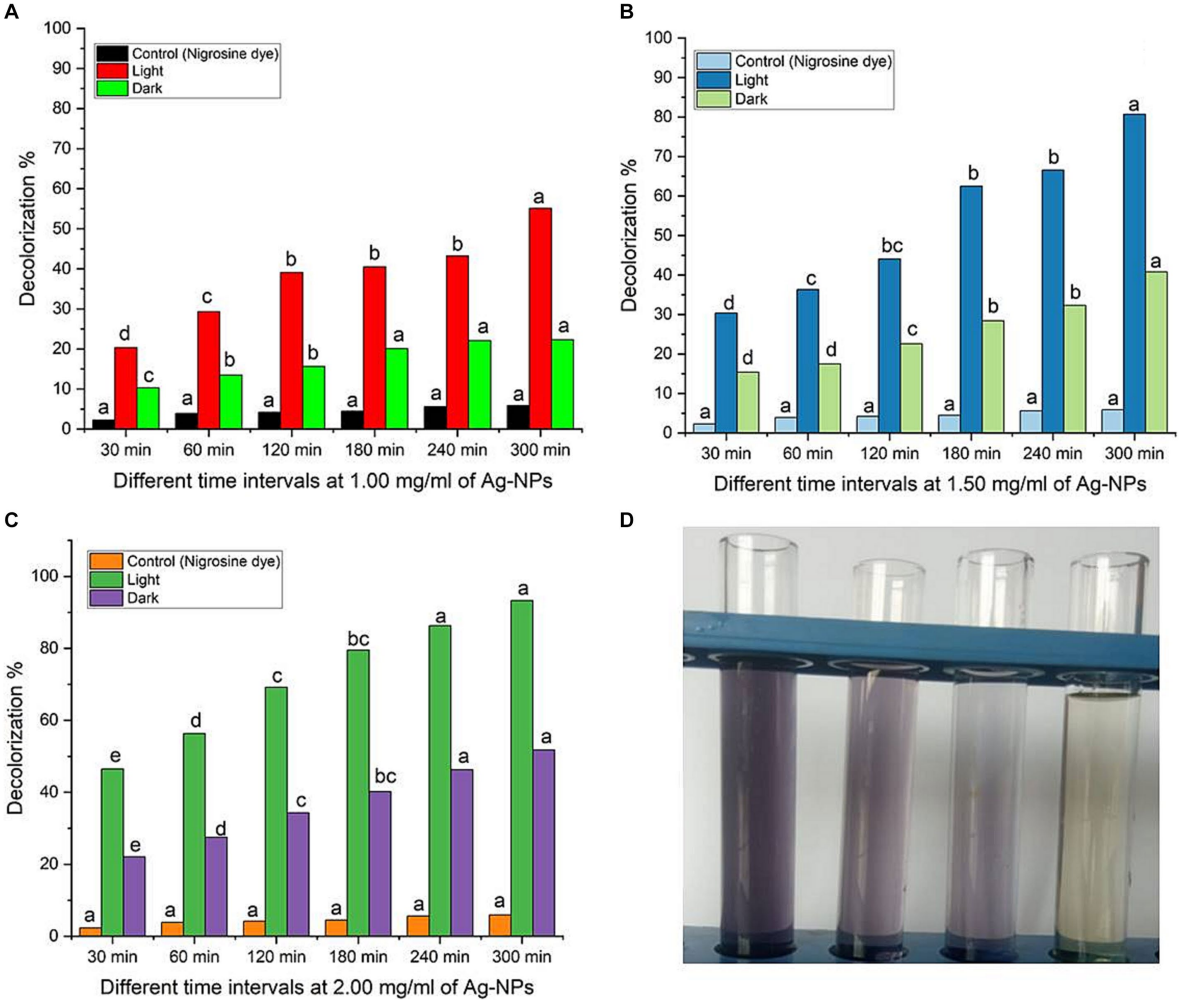
**(A–C)** Dye decolorization of nigrosine dye at varying Ag-NPs concentrations, under different stimulation conditions (dark and sunlight), and different contact times. **(D)** The image of nigrosine dye decolorization. Letters a, b, c means significance power.

Synthetic dyes are widely used in various industries, such as textiles, paper, adhesives, cosmetics, food, ink, medicines, etc. Heterocyclic azo dyes, such as nigrosine dye, are commonly released into the environment through effluents from the textile industry. It reduces the amount of oxygen on the water’s surface, impacting aquatic life. It is known to be toxic to humans and a hazard to the environment (Tripathi et al., 2021). Therefore, effluents’ nigrosine dye must degrade to eliminate its harmful effects. Ag-NPs of the appropriate size and shape possess a significant surface area-to-volume ratio, rendering them efficient catalysts for the decomposition of dyes (David and Moldovan, 2020; Rani et al., 2020). The degradation process can be carried out through photosensitization or direct application of high-energy light sources to the nanomaterials’ surface. Light activates nanoparticles, causing electrons to transition from the valence band to the conduction band. This process, known as photoexcitation, is crucial in direct photocatalytic degradation (Mehtab et al., 2022). This study aimed to explore the decolorization of nigrosine dye using Ag-NPs at various concentrations (1.0, 1.5, and 2.0 mg/mL) and contact times (30.0, 60.0, 120.0, 180.0, 240.0, and 300.0 min) under different lighting conditions. The findings indicated that the experiment’s duration and concentration influenced the catalytic activity of Ag-NPs. It’s fascinating to observe that the breakdown of Ag-NPs occurred faster when exposed to sunlight than when exposed to darkness (Figures 6A–C). Compared to the control, the decolorization percentages at 1.0 mg mL^−1^ of Ag-NPs were significantly higher under sunlight and dark conditions after 300 min. After 30 min, the decolorization percentages at 1.5 mg ml^−1^ of Ag-NPs increased to 30% ± 0.43, and after 300 min, they reached 80.7% ± 0.77. Under sunlight, a remarkable decolorization rate of 93.3% ± 0.95% was achieved within 300 min at a concentration of 2.0 mg ml^−1^ of Ag-NPs. However, in the absence of light, the decolorization rate dropped to 51.8% ± 0.83% at the same NPs concentrations and time duration. Based on the results, it was determined that a contact time of 300 min and a concentration of 2.0 mg ml − 1 of Ag-NPs yielded the most favorable outcome. Fouda et al. (2021) state that light stimulators must be present for biosynthesized MgO-NPs to degrade textile effluent effectively. According to Saied et al. (2022b) hematite (α-Fe_2_O_3_) nanoparticles were found to remove 97% of the CV dye after 150 min. The maximum dye decolorization is achieved by increasing the concentration of Ag-NPs, as their surface provides more adsorption sites (Ali and Khan, 2020). According to a study conducted by Amirtham et al. (2023), it was discovered that the CuNPs synthesized by myco exhibited different levels of degradation efficiency for various dyes. The degradation rates of the dyes varied significantly. The fast green dye degraded at a higher rate, the Congo red dye degraded at a moderate rate, and the brilliant blue dye degraded at a very low rate. Pure or single dyes’ decolorization and degradation times were compared to those of complex solutions consisting of multiple dye types or unknown compounds (Al-Askar et al., 2023). This study’s photocatalytic activities are due to sunlight stimulators activating biosynthesized Ag-NPs rather than the dark condition.

#### 3.3.1 Prospective mechanism

**SCHEME 1:**
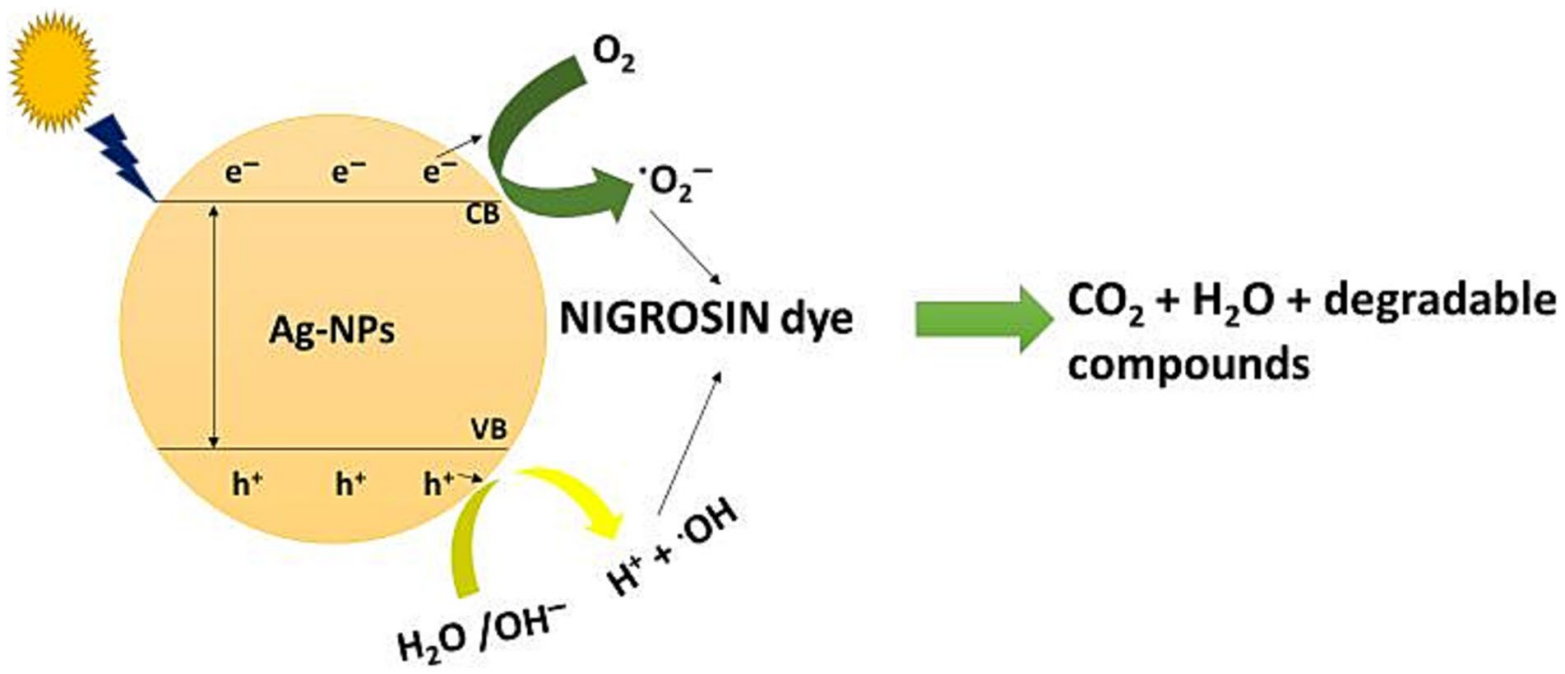
Photo-degradation mechanism of nigrosine dye by Ag-NPs synthesized by *A. hiratsukae*.

The results obtained indicate that the decolorization of nigrosine dye using AgNPs under light irradiation was more effective than that observed in dark conditions. The researchers proposed three distinct methods for removing dyes’ color using nanomaterials. One approach is to either decrease the dye in an alkaline solution or transform it into its white indigo (leuco) form. Due to the extensive surface area of nanoparticles and the wide range of dyes that can be eliminated through this process, another mechanism involves the dye being adsorbed onto the surface of nanoparticles. According to the current work, Ag-NPs may effectively remove the nigrosine dye at low light levels through the second method (Raghavendra et al., 2022). The third process is the degradation of dyes on the surface of the NPs. Considering the detection of hole electrons on the surface, which result from the electron transition induced by the SPR, this mechanism appears to be the most suitable for the Ag-NP-mediated degradation of nigrosine dye under light irradiation (Vieira et al., 2023). When electrons from the valence band (VB) are excited to the conducting band (CB) in the presence of light, electron–hole pairs [Ag (eCB and h^+^VB)] are formed (Scheme 1). When h^+^VB and H_2_O combine, hydroxyl radicals (^•^OH) and H^+^ are formed, while when O_2_ is reduced by eCB, superoxide radicals (^•^O_2_^─^) and hydrogen peroxide radicals (^•^OOH) are formed. Subsequently, the nigrosine dye reacts with the various active radical species (^•^OH, ^•^O_2_^−^, and ^•^OOH), thereby enhancing the dye’s degradation.

#### 3.3.2 Recyclability of Ag-NPs

**Figure 7:**
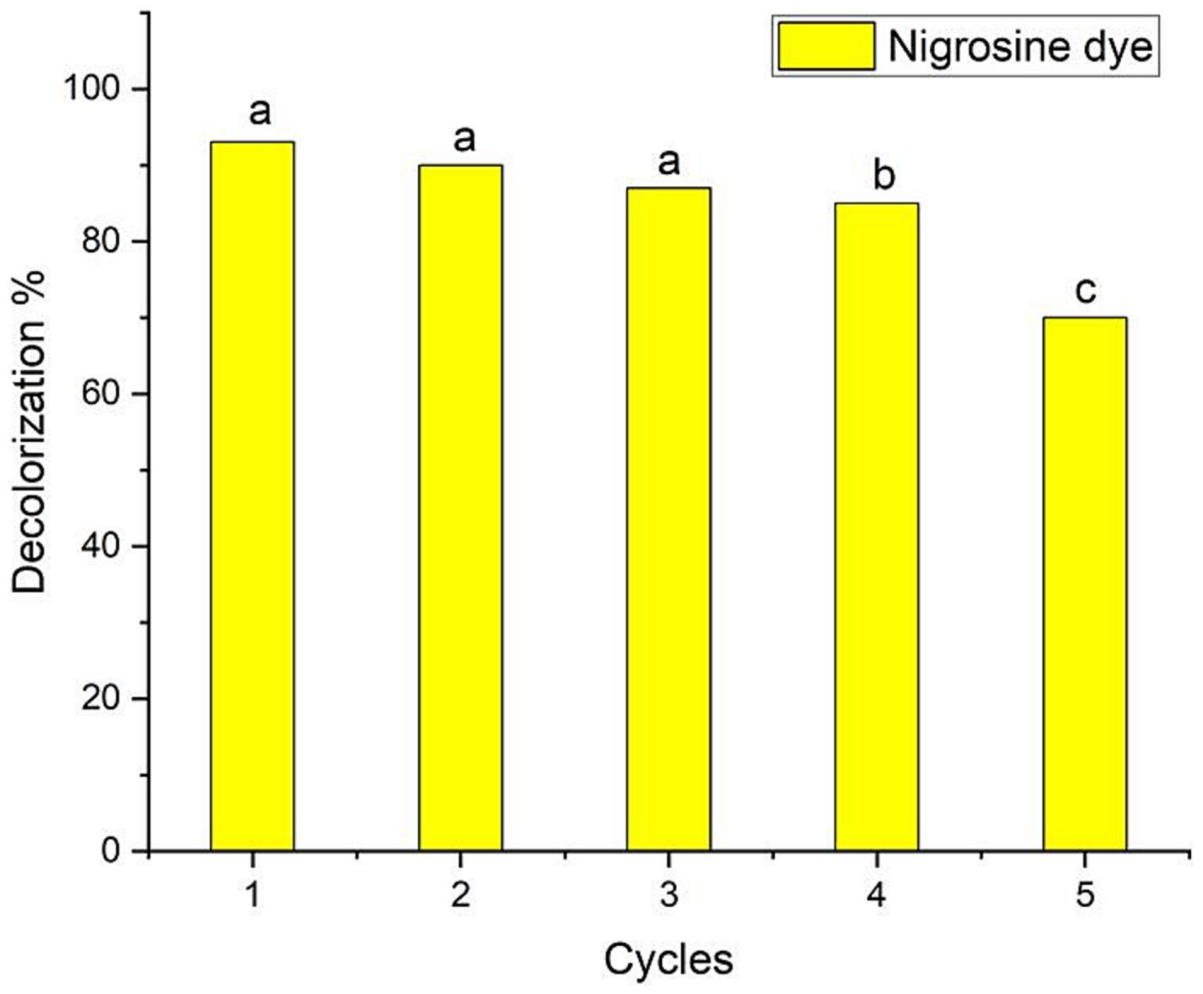
Ag-NPs’ reusability evaluation for nigrosine dye decolorization at the ideal Ag-NPs concentration (2.0 mg ml^−1^), contact time (300 min) and the presence of sunlight. Letters a, b, c means significance power.

Evaluating the photostability and reusability of the catalysts is essential to making the process economically viable (Samuel et al., 2022). The investigation in this study focused on evaluating the stability of biologically synthesized AgNPs as a biocatalyst for the recycling of nigrosine dye. The stability assessment was conducted under optimal circumstances, as depicted in Figure 6 (2.0 mg ml^−1^ biocatalyst concentration, 300 min of contact duration in the presence of photocatalytic sunlight). Following the centrifugation of the catalyst from each cycle, any residual water was eliminated through a series of three deionized water rinses, followed by a drying period of 1 h at a temperature of 120°C. Subsequently, the desiccated catalyst was utilized as a bioinoculant in the subsequent iteration. When the process was performed for four cycles, the decolorization percentages of the nigrosine dye were decreased to up to 85% ± 1.02%, as shown in the data in Figure 7. The reduced decolorization of 70% ± 0.99% is shown in cycle five. Saied et al. (2022b) reported that upon repeating the process for the fourth cycle, the decolorization percentages of crystal violet dye decreased to as low as 63.5% ± 1.04%. According to Shan et al. (2020), there was very little activity loss, and the Ag/TiO_2_/biochar composite catalysts showed good stability for up to five cycles. As stated by Paramesh et al. (2021), the recovered catalyst exhibited comparable activity for up to five cycles, and the Ag-NPs were recovered in good yield. The progressive degradation of catalyst performance was primarily caused by metal leaching concentration, adsorption of intermediate products on catalytic sites, and degradation of catalytic sites (Chen et al., 2022).

## 4 Conclusion

In conclusion, we have for the first time demonstrated a simple and cost-effective method for preparing biogenic Ag-NPs using the mycelial-free filtrate of *Aspergillus hiratsukae*. Utilizing A. hiratsukae offers benefits such as ease of cultivation. The biosynthesized AgNPs were characterized using a suite of analytical techniques, including UV–visible spectroscopy, X-ray diffraction (XRD), transmission electron microscopy (TEM), Fourier-transform infrared (FT-IR) spectroscopy, scanning electron microscopy-energy dispersive X-ray (SEM–EDX) analysis, and dynamic light scattering (DLS). The maximum SPR for biosynthesized AgNPs was observed at 420 nm. The TEM image shows particle diameters ranging from 16 to 31 nm. DLS analysis shows the size distribution, and the average particle size was 72 nm. Additionally, a crystalline nature with an average size of 37.96 nm was detected by XRD. The EDX profile of the Ag-NPs indicates the presence of the Ag element with an atomic percentage of 22.6%. The optimal conditions for the mycosynthesis of Ag-NPs were pH 10, 4 mM of the precursor, and 24 h. AgNPs also demonstrated antimicrobial activity against fungi that are unicellular, multicellular, Gram-positive, and Gram-negative. The minimum inhibitory concentrations (MICs) of the synthesized Ag-NPs ranged from 62.5 to 250 μg/mL, demonstrating remarkable antibacterial effects on *S. aureus*, *B. subtilis*, and *E. coli*. The minimum inhibitory concentrations (MIC) of *A. brasiliensis* and *C. albicans* are 500 and 1,000 μg/mL, respectively. Also, the maximum photocatalytic nigrosine dye degradation of 93% was done at 2.0 mg ml^─1^ biocatalyst concentration and 300 min of contact duration in the presence of photocatalytic sunlight. The findings reveal promising potential for employing photocatalytic dye degradation activity in environmental bioremediation, effectively removing harmful dyes from various industrial effluents.

## Data availability statement

The raw data supporting the conclusions of this article will be made available by the authors, without undue reservation.

## Author contributions

ES: Conceptualization, Data curation, Formal analysis, Investigation, Methodology, Resources, Visualization, Writing – original draft, Writing – review & editing. MA-M: Funding acquisition, Investigation, Resources, Writing – review & editing. AA: Data curation, Investigation, Writing – review & editing. BK: Resources, Validation, Visualization, Writing – review & editing. MB: Formal analysis, Writing – review & editing. OA-Q: Formal analysis, Funding acquisition, Software, Validation, Writing – review & editing. FB: Data curation, Methodology, Writing – original draft, Writing – review & editing. MSB: Methodology, Writing – review & editing. AH: Conceptualization, Data curation, Formal analysis, Investigation, Methodology, Resources, Validation, Writing – original draft, Writing – review & editing.
